# Supplementary figures and images for: Earthworm distributions are not driven by measurable soil properties. Do they really indicate soil quality?
Source: PLoS One. 2021 Aug 30;16(8):e0241945. doi: 10.1371/journal.pone.0241945 (PMC8404981; doi:10.1371/journal.pone.0241945)

Fig. S1. Typical BBN structure for the entire dataset.


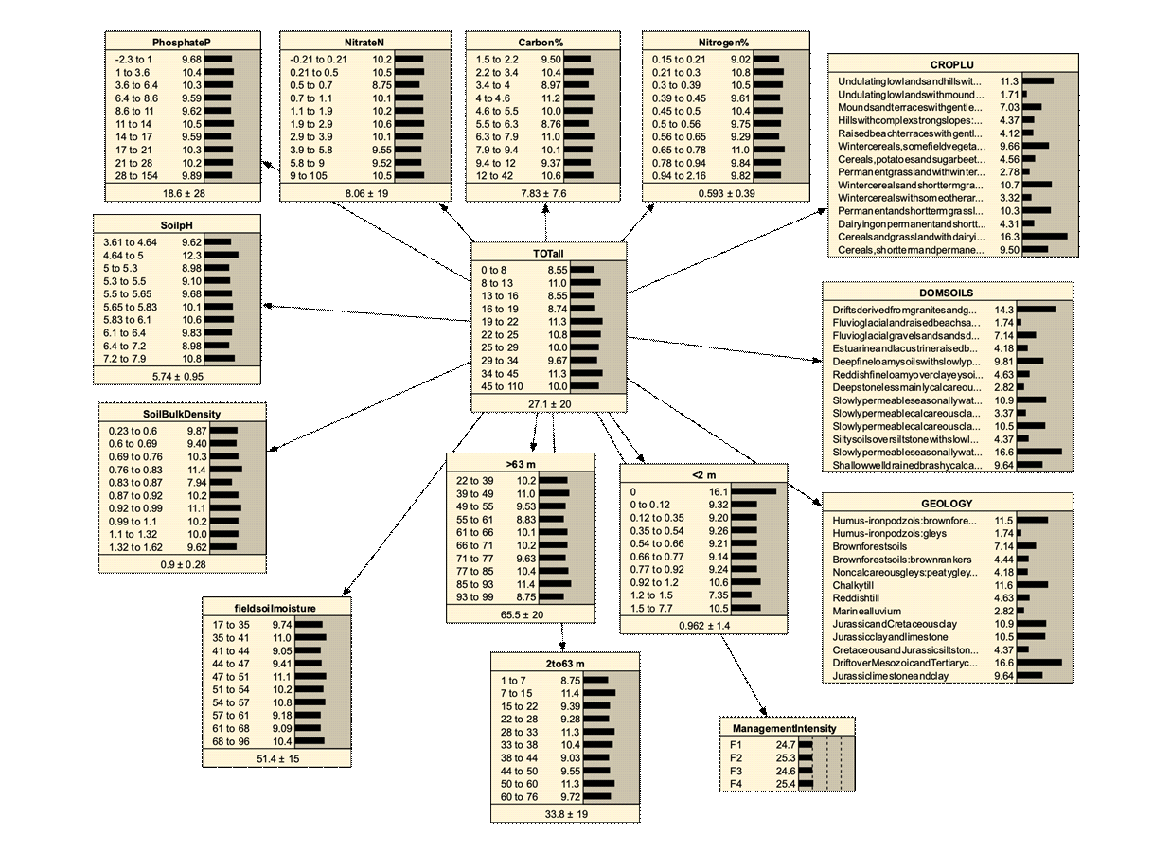

Supplement: S1 Fig — (DOCX) [file pone.0241945.s001.docx]

Table S4. Repeat ICP-OES analyses for precision


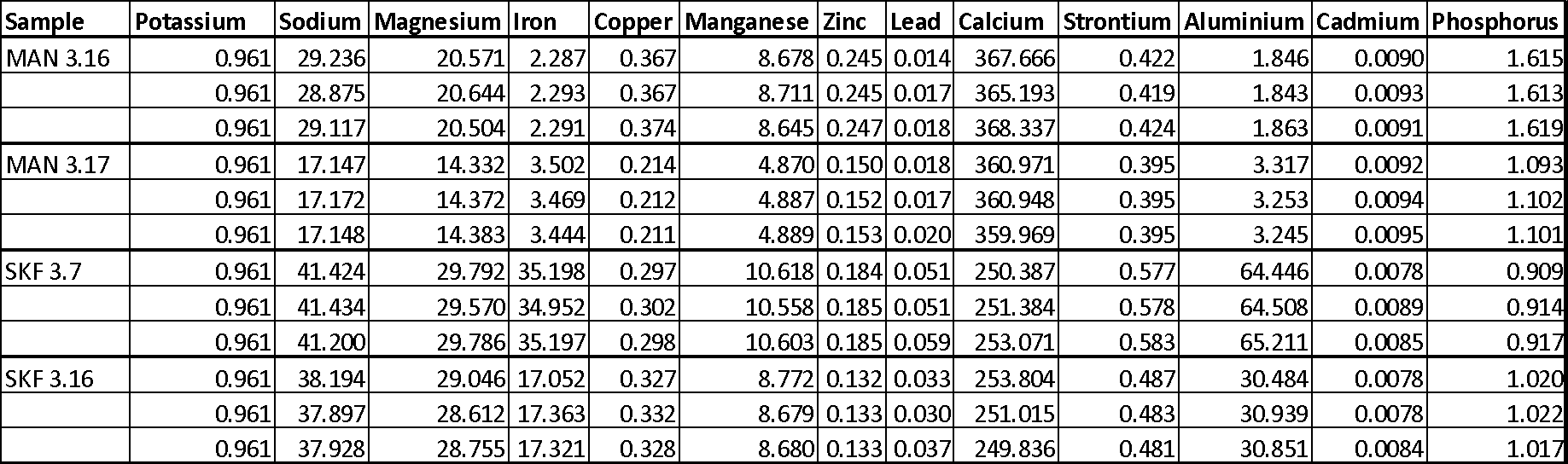

Supplement: S4 Table — (DOCX) [file pone.0241945.s005.docx]
