# Supplementary material for: Earthworm distributions are not driven by measurable soil properties. Do they really indicate soil quality?
Source: PLoS One. 2021 Aug 30;16(8):e0241945. doi: 10.1371/journal.pone.0241945 (PMC8404981; doi:10.1371/journal.pone.0241945)
Supplement: S1 Table — (DOCX) [file pone.0241945.s002.docx]

Table S1. Summary of selected previous studies that investigated the spatial distribution of earthworms in arable/pasture/savannah habitats.

| Earthworm species | Land use | Country | Climatic region | Scale of study | Analysis method | Scale of patches |  | Correlated with (-ve indicates a negative correlation) | Reference |
| --- | --- | --- | --- | --- | --- | --- | --- | --- | --- |
| *Lumbricus terrestris,*  *Allolobpphora caliginosa, Allolobophora rosea* | Arable (sugar beet, winter barley) | Germany | Temperate | 1 ha, 100 samples | Kriging and variograms | 20 – 50 m | Within field | Carbon content, aggregate density  Aggregate density (-ve) | Poier and Richter (1992) |
| Range of earthworms | Arable (Winter wheat) | UK | Temperate | 24 plots; 25 m x 5 m plots; 4 samples per plot | SADIE* | Patchy, scale not given | Within field – 2 fields sampled | C:N, shear vane | Blackshaw et al. (2007) |
| *A caliginosa*  *A rosea*  *A longa*  *L terrestris* | Arable (wheat) | Belgium | Temperate | 105 x 75 m, 100 samples | Kriging and variograms | 14 – 64 m | Within field | -  -  EC  EC(-ve) (juvenile) | Valckx et al. (2009) |
| Range of species | Arable (corn)  Hay field  Deciduous forest | Canada | Humid, continental | 25 sampling sites of 50 m x 50 m, | Other statistical methods | 16 – 21 m | Within field / forest plot | Soil moisture (forest) | Whalen (2004) |
| Range of species | Pasture (grass-clover) | Finland | Humid continental | 32 sampling sites of 25 x 25 m | Kriging and variograms | Patchy, scale not given | Within field | Soluble soil P | Nuutinen et al. (2004) |
| *L terrestris* and *A caliginosa* | Pasture | France | Temperate | 100 samples in 64 m x 64 m | Kriging and variograms | 20 – 60 m | Within field | Drainage | Cannavacciuolo et al (1998) |
| *Polypheretima elongata* | Pasture | Martinique | Tropical | 57 samples in 25 x 60 m | PCA^+^ | 20 – 30 m | Within field |  | Rossi et al (1997) |
| *Sthulmania porifera*  *Chuniodrilus zielae*  *Millsonia anomala* | Grass savannah | Ivory Coast | Tropical | 100 samples in 45 x 45 m | SADIE | 20 – 30 m | Within field |  | Rossi (2003) |
| Range of species | Savannah | Colombia | Tropical | 64 samples in 70 x 70 m | Kriging and variograms and PCA | 27 – 57 m | Within field |  | Jiménez et al (2001) |
| Range of species | Rocky pasture | Spain | Mediterranean / cold semi-arid | 42 samples in 112 x 48 m | Kriging and variograms | 22 – 100 m | Within field | *H. elisae*: sand, clay (-ve), nitrogen (-ve), carbon (-ve)  *A rosea*: sand (-ve), clay, nitrogen, carbon | Hernández et al (2007) |

* Spatial Analysis by Distance Indices methods; ^+^ Principal components analysis

Poier, KR and Richter, J, 1992. Spatial distribution of earthworms and soil properties in an arable loess soil. Soil Biology and Biochemistry 24 1601-1608.

Blackshaw, RP, Donovan, SE, Haarika, S, Bol, R, Dixon, ER, 2007. Earthworm responses to long term agricultural management practices: spatial relationships with soil properties. European Journal of Soil Biology 43 8171-8175.

Valckx, J, Cockx, L, Wauters, J, van Meirvenne, M, Govers, G, Hermy, M, Muys, B, 2009. Within-field spatial distribution of earthworm populations related to species interactions and soil apparent electrical conductivity. Applied Soil Ecology 41 315-328.

Whalen, JK, 2004. Spatial and temporal distribution of earthworm patches in corn field, hayfield and forest systems of southwestern Quebec, Canada. Applied Soil Ecology 27 143-151.

Nuutinen, V, Pitkänen, J, Kuusela, E, Widbom, T, Lohilahti, H., 1998. Spatial variation of an earthworm community related to soil properties and yield in a grass-clover field. Applied Soil Ecology 8 85-94.

Cannavacciuolo, M, Bellido, A, Cluzeau, D, Gascuel, C, Trehen, P, 1998. A geostatistical approach to the study of earthworm distribution in grassland. Applied Soil Ecology 9 345-349.

Rossi, J-P, 2003. Clusters in earthworm spatial distribution. Pedobiologia 47 490 – 496.

Rossi, J-P, Lavelle, P, Albrecht, A, 1997. Relationships between spatial pattern of the endogeic earthworm *Polypheretima elongata* and soil heterogeneity. Soil Biology and Biochemistry 29 485-488.

Jiménez, JJ, Rossi, J-P, Lavelle, P, 2001. Spatial distribution of earthworms in acid-soi savannas of the eastern plains of Colombia. Applied Soil Ecology 17 267-278.

Hernández, P, Fernández, R, Novo, M, Trigo, D, Díaz Cosin, DJ, 2007. Geostatistical and multivariate analysis of the horizontal distribution of an earthworm community in El Molar (Madrid, Spain). Pedobiologia 51 13-21.
