# Supplementary material for: Earthworm distributions are not driven by measurable soil properties. Do they really indicate soil quality?
Source: PLoS One. 2021 Aug 30;16(8):e0241945. doi: 10.1371/journal.pone.0241945 (PMC8404981; doi:10.1371/journal.pone.0241945)
Supplement: S2 Table — Numbers indicate minimum time that field has been used as pasture. “Never” indicates no use other than pasture. (DOCX) [file pone.0241945.s003.docx]

Table S2. Land use history of sampling sites according to land owners. Numbers (yrs) indicate minimum time that field has been used as pasture and area of field in hectares (Ha). “Never” indicates no use other than pasture.

|  |  | Agricultural intensification | | | |
| --- | --- | --- | --- | --- | --- |
| Sampling location | Code | Lowest | Low to medium | Medium to high | Highest |
| Craibstone, Aberdeen | CBS | F1  15 yrs  0.97 Ha | F2  Never  1.20 Ha | F3  Never  2.62 Ha | F4  15 yrs  0.97 Ha |
| Torr Organic, Dumfries | TOR | F2  40+ yrs  5.08 Ha | F1  25+ yrs  1.79 Ha | F4  3 yrs  2.08 Ha | F3  6 yrs  6.30 Ha |
| Skeffling, Yorkshire | SKF | F2  22+ yrs  1.23 Ha | F4  10 yrs  1.56 Ha | F1  22 yrs  1.83 Ha | F3  10 yrs  4.03 Ha |
| Loddington, Leicestershire | LOD | F1  22 yrs  3.42 Ha | F2  68 yrs  1.23 Ha | F3  660 yrs  3.27 Ha | F4  25+ yrs  2.62 Ha |
| Manor Farm, Somerset | MAN | F1  Never  5.81 Ha | F2  35 yrs  6.20 Ha | F3  Never  3.66 Ha | F4  35 yrs  3.75 Ha |
| Elm Farm, Berkshire | ELM | F1  30+ yrs  4.39 Ha | F2  17 yrs  5.32 Ha | F2  17 yrs  4.80 Ha | F4  20+ yrs  10.20 Ha |
| Swanage, Dorset | DST | F1  28 yrs  2.32 Ha | F2  50+ yrs  13.34 Ha | F3  35 yrs  7.48 Ha | F4  35 yrs  2.82 Ha |
