# Supplementary material for: Earthworm distributions are not driven by measurable soil properties. Do they really indicate soil quality?
Source: PLoS One. 2021 Aug 30;16(8):e0241945. doi: 10.1371/journal.pone.0241945 (PMC8404981; doi:10.1371/journal.pone.0241945)
Supplement: S3 Table — (DOCX) [file pone.0241945.s004.docx]

Table S3. Detection limits for soil analyses

| Analyte | Detection limit (mg kg^-1^ dry soil) |
| --- | --- |
| Nitrate-N | 0.015 |
| Ammonium-N | 0.051 |
| Phosphate-P | 0.060 |
| Potassium | 0.961 |
| Sodium | 0.313 |
| Magnesium | 0.023 |
| Iron | 0.063 |
| Copper | 0.025 |
| Manganese | 0.011 |
| Zinc | 0.015 |
| Lead | 0.002 |
| Calcium | 0.215 |
| Strontium | 0.001 |
| Aluminium | 0.112 |
| Cadmium | 0.008 |
| Phosphorus | 0.006 |
