# Supplementary material for: Earthworm distributions are not driven by measurable soil properties. Do they really indicate soil quality?
Source: PLoS One. 2021 Aug 30;16(8):e0241945. doi: 10.1371/journal.pone.0241945 (PMC8404981; doi:10.1371/journal.pone.0241945)
Supplement: S5 Table — (DOCX) [file pone.0241945.s006.docx]

Table S5: Earthworm and soil data set , results.

| Generalized Linear Model best fits (expressed as F-values) for selected explanatory soil variables. Species that had a best fit to the null model are not included | | | | | | | | | | | | | | |  |  |  |  |  |  |  |  |  |  |  |  |  |
| --- | --- | --- | --- | --- | --- | --- | --- | --- | --- | --- | --- | --- | --- | --- | --- | --- | --- | --- | --- | --- | --- | --- | --- | --- | --- | --- | --- |
|  |  |  |  |  |  |  |  |  |  |  |  |  |  |  |  |  |  |  |  |  |  |  |  |  |  |  |  |
| Summary of fitted Generalized Linear Models: | | | | | Summary of fitted Generalized Linear Models: | | | | | Summary of fitted Generalized Linear Models: | | | | | Summary of fitted Generalized Linear Models: | | | | | Summary of fitted Generalized Linear Models: | | | | | Summary of fitted Generalized Linear Models: | | |
|  |  |  |  |  |  |  |  |  |  |  |  |  |  |  |  |  |  |  |  |  |  |  |  |  |  |  |  |
| Predictors | Soil Bulk Density (g/cm3) | | |  | Predictors | Nitrate-N (mg/kg [dry soil]) | | |  | Predictors | Soil pH |  |  |  | Predictors | SM % mean | |  |  | Predictors | Temp mean | |  |  | Predictors | Carbon in Sample (%) | |
| Distribution | Poisson |  |  |  | Distribution | Poisson |  |  |  | Distribution | Poisson |  |  |  | Distribution | Poisson |  |  |  | Distribution | Poisson |  |  |  | Distribution | Poisson |  |
| Link function | log |  |  |  | Link function | log |  |  |  | Link function | log |  |  |  | Link function | log |  |  |  | Link function | log |  |  |  | Link function | log |  |
| GLM fitted for 11 response variables: | | | |  | GLM fitted for 14 response variables: | | | |  | GLM fitted for 13 response variables: | | | |  | GLM fitted for 11 response variables: | | | |  | GLM fitted for 11 response variables: | | | |  | GLM fitted for 13 response variables: | | |
| Response | Type | F |  |  | Response | Type | F |  |  | Response | Type | F |  |  | Response | Type | F |  |  | Response | Type | F |  |  | Response | Type | F |
| Al-chl | quadratic | 19.1 |  |  | Al-chl | quadratic | 184.2 |  |  | Al-chl | quadratic | 150.8 |  |  | Al-chl | quadratic | 191.2 |  |  | Al-chl | quadratic | 71.4 |  |  | Al-chl | quadratic | 35.5 |
| Ad-esi | quadratic | 8.9 |  |  | Ad-esi | quadratic | 9.9 |  |  | Ad-esi | quadratic | 8.9 |  |  | Ad-esi | quadratic | 6.9 |  |  | Ad-esi | quadratic | 9.6 |  |  | Ad-esi | quadratic | 6 |
| Ap-noc | linear | 4.9 |  |  | Ap-cal | quadratic | 8.9 |  |  | Ap-cal | quadratic | 14.5 |  |  | Ap-cal | linear | 9.4 |  |  | Ap-cal | quadratic | 11.6 |  |  | Ap-cal | quadratic | 2.9 |
| Ap-lim | quadratic | 28.5 |  |  | Ap-lim | quadratic | 6.9 |  |  | Ap-lim | quadratic | 79.5 |  |  | Ap-lim | quadratic | 63.6 |  |  | Ap-noc | linear | 4.9 |  |  | Ap-noc | linear | 5.3 |
| Ap.ros | quadratic | 9.8 |  |  | Ap-lon | quadratic | 40.1 |  |  | Ap-lon | quadratic | 36 |  |  | Ap-lon | quadratic | 24.3 |  |  | Ap-lim | quadratic | 42.1 |  |  | Ap-lim | quadratic | 33.1 |
| Ds-rub | quadratic | 3.1 |  |  | Ap.ros | linear | 21.3 |  |  | Ap.ros | quadratic | 2.6 |  |  | Ap.ros | quadratic | 26 |  |  | Ap-lon | quadratic | 40.9 |  |  | Ap-lon | quadratic | 10.3 |
| L-cas | quadratic | 30 |  |  | Ds-rub | linear | 12.6 |  |  | El-tet | quadratic | 7.7 |  |  | Ds-rub | quadratic | 3.1 |  |  | L-cas | quadratic | 9.4 |  |  | Ap.ros | linear | 6.2 |
| L-rub | quadratic | 9.3 |  |  | L-cas | quadratic | 24 |  |  | L-cas | quadratic | 6.1 |  |  | L-cas | quadratic | 11.2 |  |  | L-fes | quadratic | 19.8 |  |  | Ds-rub | quadratic | 2.5 |
| M-mul | quadratic | 4.5 |  |  | L-fes | quadratic | 11.1 |  |  | L-fes | quadratic | 20.7 |  |  | L-ter | quadratic | 4.7 |  |  | M-mul | quadratic | 24.3 |  |  | L-cas | quadratic | 11.1 |
| O-tyr | quadratic | 3.6 |  |  | L-rub | quadratic | 6.4 |  |  | L-rub | linear | 17.9 |  |  | O-tyr | quadratic | 6.2 |  |  | O-cya | linear | 8.8 |  |  | L-fes | quadratic | 3.4 |
| S-mam | quadratic | 12 |  |  | M-mul | quadratic | 2.8 |  |  | M-mul | quadratic | 9.5 |  |  | S-mam | quadratic | 2.3 |  |  | S-mam | quadratic | 18.9 |  |  | M-mul | quadratic | 7.4 |
|  |  |  |  |  | O-cya | linear | 3.5 |  |  | O-cya | linear | 5 |  |  |  |  |  |  |  |  |  |  |  |  | O-tyr | quadratic | 3.1 |
|  |  |  |  |  | O-tyr | quadratic | 2.4 |  |  | O-tyr | quadratic | 3.4 |  |  |  |  |  |  |  |  |  |  |  |  | S-mam | quadratic | 19.2 |
|  |  |  |  |  | S-mam | quadratic | 17.2 |  |  |  |  |  |  |  |  |  |  |  |  |  |  |  |  |  |  |  |  |
