# Supplementary material for: Earthworm distributions are not driven by measurable soil properties. Do they really indicate soil quality?
Source: PLoS One. 2021 Aug 30;16(8):e0241945. doi: 10.1371/journal.pone.0241945 (PMC8404981; doi:10.1371/journal.pone.0241945)
Supplement: S6 Table — a. Sensitivity of BBN for epigeic earthworms. See Fig 3 and Table 1 of main paper for how to interpret these tables. b. Sensitivity of BBN for endogeic earthworms. See Fig 3 and Table 1 of main paper for how to interpret these tables. c. Sensitivity of BBN for anecic earthworms. See Fig 3 and Table 1 of main paper for how to interpret these tables. (DOCX) [file pone.0241945.s007.docx]

Table S6a. Sensitivity of BBN for epigeic earthworms. See Fig. 3 and Table 1 of main paper for how to interpret these tables

Table S6b. Sensitivity of BBN for endogeic earthworms. See Fig. 3 and Table 1 of main paper for how to interpret these tables

Table S6c. Sensitivity of BBN for anecic earthworms. See Fig. 3 and Table 1 of main paper for how to interpret these tables
