# Supplementary material for: Earthworm distributions are not driven by measurable soil properties. Do they really indicate soil quality?
Source: PLoS One. 2021 Aug 30;16(8):e0241945. doi: 10.1371/journal.pone.0241945 (PMC8404981; doi:10.1371/journal.pone.0241945)
Supplement: S8 Table — a. Results from the Bayesian Belief Networks developed for each individual farm for epigeic earthworms. See Table 2, main manuscript, for further information. b. Results from the Bayesian Belief Networks developed for each individual farm for endogeic earthworms. (See Table 2, main manuscript, for further information). c. Results from the Bayesian Belief Networks developed for each individual farm for anecic earthworms. See Table 2, main manuscript, for further information. (DOCX) [file pone.0241945.s009.docx]

Table S8a. Results from the Bayesian Belief Networks developed for each individual farm for epigeic earthworms. See Table 2, main manuscript, for further information.

Table S8b. Results from the Bayesian Belief Networks developed for each individual farm for endogeic earthworms. See Table 2, main manuscript, for further information.

Table S8c. Results from the Bayesian Belief Networks developed for each individual farm for anecic earthworms. See Table 2, main manuscript, for further information.
