# Supplementary material for: Earthworm distributions are not driven by measurable soil properties. Do they really indicate soil quality?
Source: PLoS One. 2021 Aug 30;16(8):e0241945. doi: 10.1371/journal.pone.0241945 (PMC8404981; doi:10.1371/journal.pone.0241945)
Supplement: S9 Table — a. Variogram parameters for the seven sites for epigeic earthworms. See Table 3, main manuscript for details. b. Variogram parameters for the seven sites for endogeic earthworms. See Table 3, main manuscript for details. c. Variogram parameters for the seven sites for anecic earthworms. See Table 3, main manuscript for details. (DOCX) [file pone.0241945.s010.docx]

Table S9a. Variogram parameters for the seven sites for epigeic earthworms. See Table 3, main manuscript for details.

Table S9b. Variogram parameters for the seven sites for endogeic earthworms. See Table 3, main manuscript for details.

Table S9c. Variogram parameters for the seven sites for anecic earthworms. See Table 3, main manuscript for details.
